# The oncometabolite 2-hydroxyglutarate produced by mutant IDH1 sensitizes cells to ferroptosis

**DOI:** 10.1038/s41419-019-1984-4

**Published:** 2019-10-07

**Authors:** Tian-Xiang Wang, Jun-Yun Liang, Cheng Zhang, Yue Xiong, Kun-Liang Guan, Hai-Xin Yuan

**Affiliations:** 1 The Fifth People’s Hospital of Shanghai and the Molecular and Cell Biology Research Lab of the Institutes of Biomedical Sciences, Fudan University, Shanghai, China; 2 Department of Biochemistry and Biophysics, Lineberger Comprehensive Cancer Center, University of North Carolina at Chapel Hill, Chapel Hill, North Carolina USA; 3 Department of Pharmacology and Moores Cancer Center, University of California San Diego, La Jolla, California USA

**Keywords:** Cancer, Cell death

## Abstract

Ferroptosis is a non-apoptotic form of cell death characterized by the iron-dependent lipid peroxidation and is implicated in several human pathologies, such as tissue ischemia, neurodegeneration, and cancer. Ferroptosis appears to be high cell-context dependent and the regulation of ferroptosis by physiological or pathological conditions are unclear. Here, we report that tumor-derived *IDH1* mutation sensitizes cells to ferroptosis. Deletion of the mutant *IDH1* allele in *IDH1* heterozygous tumor cells or pharmacological inhibition of mutant IDH1 to produce the oncometabolite D-2-hydroxyglutarate (D-2-HG) confers resistance to erastin-induced ferroptosis. Conversely, ectopic expression of mutant IDH1 or treatment of cells with cell-permeable D-2-HG promotes the accumulation of lipid reactive oxygen species (ROS) and subsequently ferroptosis. Mechanistically, mutant IDH1 reduces the protein level of the glutathione peroxidase 4 (GPX4), a key enzyme in removing lipid ROS and ferroptosis, and promotes depletion of glutathione. Our results uncover a new role of mutant *IDH1* and 2-HG in ferroptosis.

## Introduction

Ferroptosis is a recently identified non-apoptotic form of regulated cell death that is prone to happen in tumor cells bearing *Ras* gene mutation^1^ or highly transformed tumor cells^2^. Ferroptosis is distinct from apoptosis or necroptosis based on the fact that caspase or RIPK1 inhibitors do not hinder ferroptosis process. Ferroptosis also displays unique morphological features such as shrunken mitochondria and increased mitochondrial membrane density^3^. Although the physiological functions of ferroptosis remains elusive, much efforts have been taken in recent years to elucidate the mechanisms underlying ferroptosis. It is believed that excessive accumulation of lipid peroxide (lipid ROS), generated by the family of lipoxygenases, is a critical cause leading to ferroptosis^4^. This links ferroptosis with the breakdown of cellular redox homeostasis maintained by glutathione and glutathione peroxidase 4 (GPX4), the only enzyme in mammalian cells that could eliminate lipid ROS using reduced glutathione (GSH) as a substrate. Accordingly, compounds that inhibit the lipoxygenases such as Nordihydroguaiaretic acid (NDGA) and zileuton are effective in suppressing ferroptosis^5^. On the other hand, compounds that inhibit cystine-glutamate antiporter (system X*c*^–^) and subsequently lower GSH level (such as erastin) or that inhibit GPX4 activity (such as RSL3) strongly induce ferroptosis.

Besides system X*c*^−^ (*SLC7A11*) and *GPX4*, several other genes have also been reported to influence cells’ sensitivity to ferroptosis including *ACSL4*, *TP53* and *GLS2*^6–8^. These studies linked ferroptosis to multiple cellular processes like iron homeostasis, redox homeostasis, lipid metabolism, and glutaminolysis. However, the fact that ferroptosis is highly dependent on cell-type indicates the complex mechanisms underlying ferroptosis. Given highly transformed and drug-resistant tumor cells are prone to ferroptosis, it is of great importance to understand the mechanism underlying ferroptosis and apply it to personalized anti-cancer strategies and some other diseases such as I/R injury and even neuron degenerative diseases^9–11^.

Hot-spot mutations of the metabolic enzyme IDH1/2 (isocitrate dehydrogenases 1 and 2) are observed in several types of cancers including secondary glioblastomas (GBMs)^12^, acute myeloid leukemia (AML)^13^, chondrosarcoma^14–16^ and cholangiocarcinoma^17,18^. The high frequent mutations on the single residue (R132 in *IDH1*, R172 or R140 in *IDH2*) have caught broad interest in its function in promoting tumor development. Disease-associated IDH1/2 mutations not only abolish their enzyme activity in converting isocitrate to α-KG, but also endow the mutant protein with a new activity to convert α-KG to (D)-2-hydroxyglutarate (2-HG) using NADPH as co-factor [reviewed in ref. ^19^]. In tumor cells bearing IDH mutations, 2-HG can be accumulated at concentrations of millimolar level^20^, at which it competitively inhibits multiple α-KG/Fe(II)-dependent dioxygenases^21,22^, consequently interfering many relevant processes as epigenetic regulation, genetic instability, T cell differentiation and tumor immunity [reviewed in ref. ^19^]. Interestingly, 2-HG was also found to suppress cell growth through binding and inhibiting ATP synthase, a function that α-KG also executes, suggesting complex relationship between the two metabolites^23,24^. In addition, elevated 2-HG has also been observed in tumor cells lacking an IDH mutation and even in non-tumorigenic heart during ischemic preconditioning and hematopoietic stem cells after disruption of the respiratory chain [reviewed in ref. ^19^]. These studies demonstrate 2-HG is involved in pathological conditions not limited to cancer.

Here, we examined the relationship between mutant IDH1 and ferroptosis sensitivity in response to erastin. We find that high level of 2-HG resulting from *IDH1*^*R132C*^ mutation sensitizes cells to erastin-induced ferroptosis. In detail, *IDH1*^*R132C*^ mutation and its metabolic product 2-HG could decrease the protein level of GPX4 and result in a rapid exhaustion of glutathione upon erastin. Our results present a novel role of tumor-derived IDH1 mutation and oncometabolite 2-HG in ferroptosis.

## Materials and methods

### Antibodies, plasmid, and chemicals

Antibodies against Flag (ShanghaiGenomics), β-actin (Genescript), GPX4 (Abcam), ACSL4 (Proteintech), ERK (CST), p-ERK (CST), NRF2 (Abcam) were purchased commercially.

Full-length cDNA of *D2HGDH* and *IDH1* was amplified by PCR and cloned into indicated pBabe and pQCXIH. Point mutations for *IDH1* were generated by site-directed mutagenesis and verified by Sanger sequencing.

AG-120 (CSNpharm), IDH-889 (DC Chemicals), erastin (MedChemExpress, MCE), RSL3 (MCE), Deferoxamine mesylate (MCE), Ferrostatin-1 (Selleck Chemicals), (2 R)-2-Hydroxyglutaric Acid Octyl Ester Sodium Salt, and (2S)-2-Hydroxyglutaric Acid Octyl Ester Sodium Salt (Toronto Research Chemicals) were purchased commercially.

### Cell culture, transfection, and stable cell lines generation

HEK293T, HT-1080 and KYSE-170 cells were purchased from the American Type Culture Collection (ATCC). HEK293T and HT-1080 cells were cultured in DMEM (Invitrogen) supplemented with 5% FBS (Gibco), 100 unit/mL penicillin, and 100 mg/mL streptomycin (Gibco). KYSE-170 cells were cultured in RPMI 1640 medium (Gibco) with 10% FBS, 100 unit/mL penicillin, and 100 mg/mL streptomycin.

Cell transfection was carried out by Lipofectamine 2000 according to the manufacturer’s protocol (Invitrogen).

Cells stably expressing the indicated proteins were established by standard retroviral infection, and selected in 2 mg/mL puromycin (Ameresco) or 50 mg/mL hygromycin B (Ameresco) for 7 days. The mutant IDH1 allele knocked out HT-1080(*IDH1*^+/−^) cells were generated previously by TALEN technology.

### Cell viability assay

Cells were seeded into 12-well plates and incubated with the indicated treatments. Subsequently, cells were digested with trypsin and collected for trypan blue staining. Cell viability was counted and calculated by using an automated cell counter (Count Star, IC 1000). Cell viability under test conditions is reported as a percentage relative to the negative control treatment.

### Measurement of intracellular glutathione level

Cells were seeded into 6-well dishes at a concentration of 400,000 cells/well. After one day, cells were treated with erastin for the indicated times and harvested by trypsinization. Cell numbers were counted by using an automated cell counter (Count Star, IC 1000). Intracellular glutathione level was detected using the Total Glutathione Assay Kit (Beyotime, S0052) following the manufacture’s instructions.

### Cellular lipid ROS assay

Cells were seeded into 6-well dishes at a concentration of 400,000 cells/well. After one day, cells were treated with erastin for the indicated times. After treatment, cells were stained with C11-BODIPY 581/591 (Thermo Fisher Scientific, D3861) for 30 min at 37 °C and then harvested by trypsinization. Cells were re-suspended in PBS and strained through a 40-μm cell strainer (BD Falcon), and then analyzed cells using flow cytometer (Accuri C6, BD Biosciences) equipped with 488 nm laser for excitation. Data were collected from the FL1. A minimum of 10,000 cells were analyzed per condition.

### Metabolite extraction and GC-MS analysis

Confluent cells in 6-well plated were homogenized in 0.5 mL of chilled 80% (v/v) methanol. The samples were centrifuged at 12,000 rpm for 10 min and the supernatants were transferred to a high recovery glass sampling vial (CNW, VAAP-31509-1232-100) to vacuum dry at room temperature. The residue was re-suspended with 30 μL pyridine containing 20 mg/mL methoxyamine hydrochloride (Sigma-Aldrich, 226904) at 37 °C overnight and further derivatized with 20 μL of N-tert-Butyldimethylsilyl-N-methyltrifluoroacetamide (Sigma-Aldrich, Cat#: 394882) at 70 °C for 30 min. Then 1 μL aliquot of the derivatized sample was injected into Agilent 7890 A gas chromatography coupled with Agilent 5975 C mass spectrometer. Separation was achieved on a HP-5ms fused-silica capillary column with the helium as the carrier gas at a constant flow rate of 1 mL/min through the column.

### Statistical analysis

Statistical analyses were performed using GraphPad Prism 6.0c. All data shown represent the results obtained from triplicated independent experiments with standard deviation of the mean (mean ± SD). The sample sizes were chosen to allow for statistical significance testing assuming a major effect and a small variation. The variance was similar between the compared groups. The *p* values were calculated with two-tailed unpaired Student’s *t*-test or one-way ANOVA with Dunnett’s multiple comparisons test or Bonferroni’s multiple comparisons test as indicated in corresponding figure legends. *p* < 0.05 were considered statistically significant.

## Results

### Mutant IDH1 promotes erastin-induced ferroptosis

**Fig. 1:**
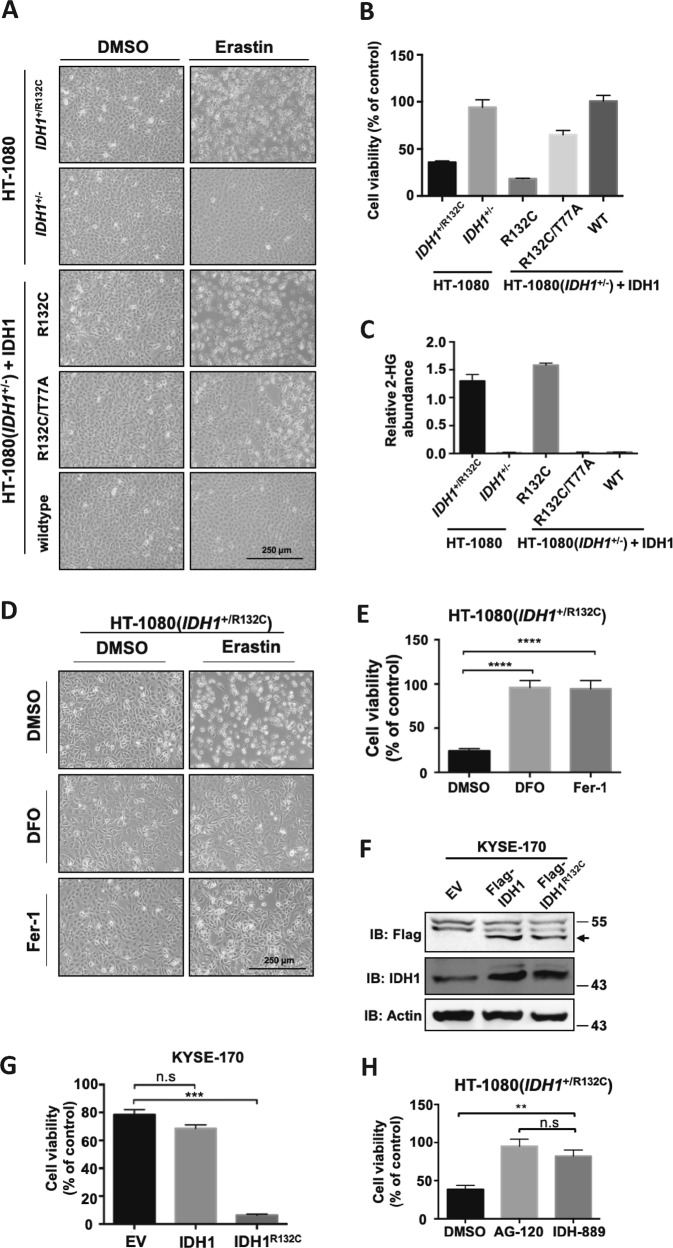
Mutant IDH1 promotes erastin-induced ferroptosis. **a** IDH1^R132C^ mutation promotes erastin-induced ferroptosis. HT-1080 cells with indicated IDH1 genotypes were treated with erastin (10 μM) for 12 h, and microscopically photographed. **b** Cell viability in (**a**) were assayed by trypan blue staining. **c** Endogenous 2-HG levels of HT-1080 cells with different IDH1 genotypes in (**a**) were determined by GC-MS. **d** Ferroptosis induction in HT-1080 cells. HT-1080 cells were treated with erastin, DFO, and Fer-1 as indicated for 12 h, and microscopically photographed. **e** Cell viability in (**d**) were assayed by trypan blue staining (*n* = 3; ****p < 0.0001 vs. DMSO group, Dunnett’s multiple comparisons test). **f** Expression of Flag-IDH1 or Flag-IDH1^R132C^ in KYSE-170 cells were detected by Western blot. **g** IDH1^R132C^ mutation promotes erastin-induced ferroptosis in KYSE-170 cells. KYSE-170 cells expressing Flag-IDH1 or Flag-IDH1^R132C^ were treated with erastin for 16 h and cell viability was assayed by trypan blue staining (*n* = 3; ***p < 0.001 vs. EV group, Dunnett’s multiple comparisons test). **h** Pharmacological inhibition of mutant IDH1 reduces cells’ sensitivity to erastin. HT-1080 cells were treated with AG-120 or IDH-889 for 12 h, and then with erastin for additional 12 h. Cell viability were assayed by trypan blue staining (*n* = 3; **p < 0.01 and ***p < 0.001, Bonferroni’s multiple comparisons test)

HT-1080 is a model cell line extensively used for ferroptosis study because of its high sensitivity to ferroptosis-inducing compounds, such as erastin and RSL3^25^. HT-1080 cells harbor Ras mutation that makes cells prone to ferroptosis. Notably, HT-1080 also bears a heterozygous R132C mutation in IDH1 [referred to as HT1080(*IDH1*^+/R132C^)^26^]. No report has linked IDH1 mutation with ferroptosis by now. We previously generated HT-1080 cell lines with the mutant IDH1 allele knocked out [referred to as HT1080(*IDH1*^+/−^)] and then putting back wild-type, R132C single mutant or R132C/T77A double mutant of IDH1 gene. Among them, R132C/T77A double mutant inactivates the activity of mutant IDH1 to produce D-2-HG^27^. We performed ferroptosis assays in these isogenic cell lines following treatment with erastin. Surprisingly, deletion of *IDH1*^R132C^ mutant allele significantly reduced erastin-induced ferroptosis (Fig. 1a). Compared to the parental HT1080(*IDH1*^+/R132C^) cells, cell viability was increased by 2.5 folds in HT1080(*IDH1*^+/−^) cells (Fig. 1b). Re-expression of the R132C mutant of *IDH1*, accumulated D-2-HG (Fig. 1c) and restored cells’ sensitivity to erastin (Fig. 1b), indicating the production of 2-HG is required for HT-1080 cells’ sensitivity to erastin. Though significantly rescuing cell death compared with R132C mutant, re-expression of R132C/T77A double mutant still partially restored cells’ sensitivity to erastin (Fig. 1b). This data indicated besides 2-HG, IDH1 mutant may have additional mechanism to promote ferroptosis. We confirmed that erastin-induced cell death was ferroptosis using an iron chelator, deferoxamine (DFO), and a ferroptosis inhibitor, Ferrostatin-1 (Fig. 1d, e).

To further confirm the role of *IDH1*^R132C^ mutation in enhancing erastin-induced ferroptosis, we overexpressed wild-type or R132C mutant *IDH1* in KYSE-170 esophagus tumor cells which contain two wild-type *IDH1* alleles (Fig. 1f). Consistently, overexpression of IDH1^R132C^ promoted erastin-induced ferroptosis while wide type IDH1 overexpression exerted no effect on cells’ sensitivity to erastin (Fig. 1g). We also treated HT-1080 cells with two small molecules that specifically inhibit mutant IDH1, AG-120 (Ivosidenib)^28^ and IDH-889^29^, and found that both inhibitors reduced cells’ sensitivity to erastin (Fig. 1h). Together, these data demonstrate that IDH1^R132C^ mutation promotes cells’ sensitivity to erastin-induced ferroptosis.

### Mutant IDH1 enhances erastin-induced lipid ROS accumulation

**Fig. 2:**
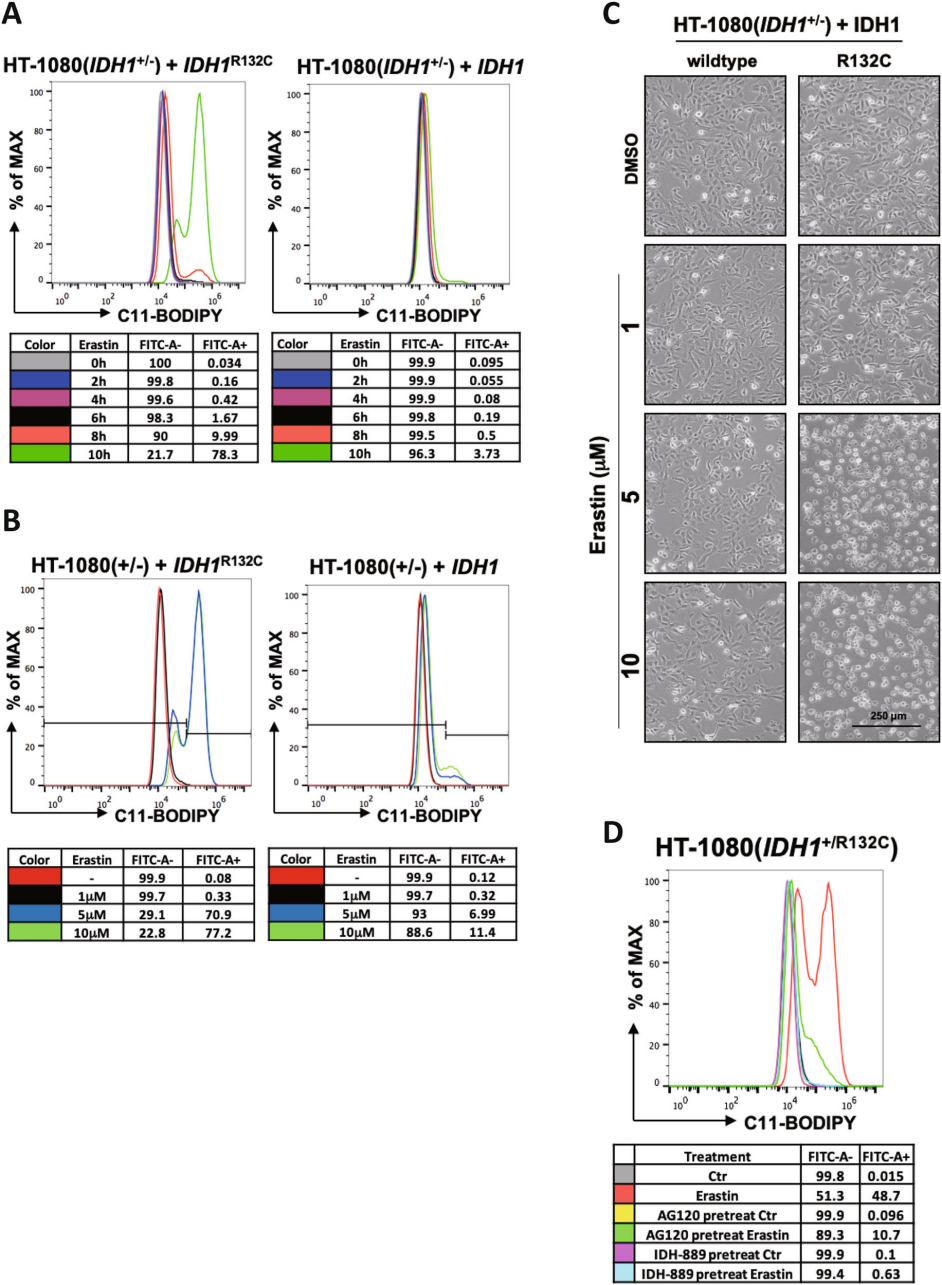
Mutant IDH1 enhances erastin-induced lipid ROS accumulation. **a** IDH1^R132C^ mutation enhances erastin-induced lipid ROS accumulation in a time-dependent manner. HT-1080(*IDH1*^+/−^) cells expressing ectopic IDH1 or IDH1^R132C^ were treated with erastin (10 μM) for indicated time and the accumulation of lipid ROS was assessed by C11 BODIPY 581/591 staining coupled with flow cytometry analysis. **b** Mutant IDH1 enhances erastin-induced lipid ROS accumulation in a concentration-dependent manner. HT-1080(IDH1^+/−^) cells expressing ectopic IDH1 or IDH1^R132C^ were treated with different concentrations of erastin for 10 h and the accumulation of lipid ROS within cells was assessed by C11 BODIPY 581/591 staining coupled with flow cytometry analysis. **c** Cell state in (**b**) was captured by microscope. **d** Mutant IDH1 inhibitors inhibit erastin-induced lipid ROS accumulation. HT-1080(IDH1^+/R132C^) cells were treated with mutant IDH1 inhibitors AG-120 or IDH-889 for 12 h, and then with erastin for additional 10 h. The accumulation of lipid ROS within cells was assessed by C11 BODIPY 581/591 staining coupled with flow cytometry analysis

Excessive accumulation of lipid ROS is a critical cause of ferroptosis which could be detected by using fluorescent radio-probe C11 BODIPY 581/591. To determine whether mutant IDH1 could promote cells’ sensitivity to erastin by increasing lipid ROS, we measured the lipid ROS levels in HT-1080 cells with different genotypes of *IDH1*. As shown in Fig. 2a, lipid ROS began to accumulate at 8 h in HT-1080(*IDH1*^+/−^) cells ectopically expressing *IDH1*^R132C^ and rapidly increased at 10 h after erastin addition. At 10 h, approximately 80% of the cells showed high level of lipid ROS. In contrast, there was no significant increase of lipid ROS in HT-1080(*IDH1*^+/−^) cells ectopically expressing wide-type *IDH1* in the same duration.

We next determined the effect of *IDH1* mutation to different doses of erastin. We found that 5 μM of erastin strongly induced lipid ROS accumulation in cells expressing *IDH1* mutant, but not in cells expressing wild-type *IDH1*. At a lower dose of erastin (1 μM), there was no significant accumulation of lipid ROS (Fig. 2b). Consistent with this, 1 μM of erastin did not induce cell death while 5 μM of erastin was able to cause ferroptosis in cells expressing R132C mutant, but not wild-type *IDH1* (Fig. 2c). In addition, IDH1 mutant inhibitors AG-120 and IDH-889 also suppressed erastin-induced lipid ROS accumulation in HT1080(*IDH1*^+/R132C^) cells (Fig. 2d). Collectively, these data indicate that mutant *IDH1* promotes erastin-induced ferroptosis through increasing lipid ROS accumulation in a catalytic-dependent manner.

### D-2-HG promotes erastin-induced ferroptosis

**Fig. 3:**
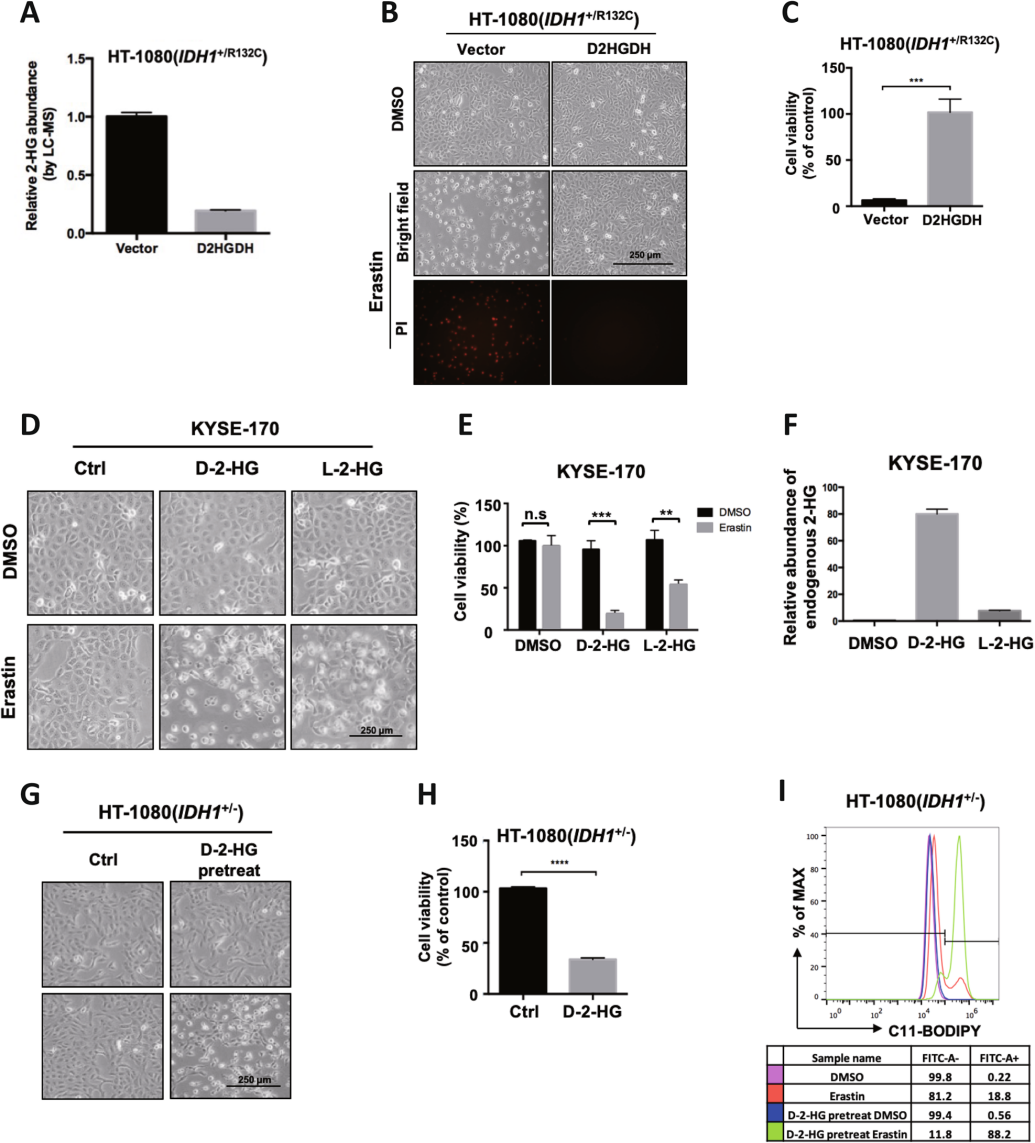
D-2-HG promotes erastin-induced ferroptosis. **a** Overexpression of D2HGDH inhibits 2-HG accumulation. Cellular 2-HG level in HT-1080 cells with empty vector or D2HGDH overexpression were determined by LC-MS. **b** Clearance of D-2-HG by D2HGDH overexpression inhibits erastin-induced ferroptosis. HT-1080 cells with empty vector or D2HGDH overexpression were treated with erastin for 12 h and cell state was captured by microscope. Dead cells were stained by propidium iodide (PI). **c** Cell viability in (**b**) were assayed by trypan blue staining g (*n* = 3; **p < 0.01, two-tailed unpaired *t*-test). **d** 2-HG promotes erastin-induced ferroptosis. KYSE-170 cells were treated with D-2-HG or L-2-HG for 24 h, and then cells were treated with erastin for additional 16 h. Cell states were captured by microscope. **e** Cell viability in (**d**) were assayed by trypan blue staining (*n* = 3; **p < 0.01 and ***p < 0.001, two-tailed unpaired *t*-test). **f** Endogenous 2-HG level of KYSE-170 cells in (**d**) were determined by GC-MS. **g** 2-HG promotes erastin-induced ferroptosis. HT-1080 (*IDH1*^+/−^) cells were treated with D-2-HG for 24 h and then with erastin for subsequent 12 h, followed by microscopic photograph. **h** Cell viability in (**g**) were assayed by trypan blue staining (*n* = 3; ****p < 0.0001, two-tailed unpaired *t*-test). **i** D-2-HG treatment enhances erastin-induced lipid ROS accumulation. HT-1080 (*IDH1*^+/−^) cells were first treated with D-2-HG for 24 h and then erastin for 10 h. Lipid ROS accumulation was assessed by C11 BODIPY 581/591 staining coupled with flow cytometry analysis

IDH1^R132C^ mutant confers a neomorphic enzymatic gain-of-function to convert α-KG to D-2-HG. High level of 2-HG was detected in cells expressing mutant *IDH1*^R132C^ (Fig. 1c). Ectopic expression of R132C mutant in HT-1080(*IDH1*^+/−^) cells, but not the R132C/T77A double mutant which inactivates the production of 2-HG re-sensitized cells to erastin-induced ferroptosis. To directly demonstrate the role of 2-HG in enhancing ferroptosis, we overexpressed D-2-HG dehydrogenase (D2HGDH), which catalyzes the oxidation of D-2-HG to α-KG and thus reduces D-2-HG level in HT-1080(*IDH1*^+/R132C^) cells (Fig. 3a). In line with this, ferroptosis induced by erastin was inhibited by D2HGDH overexpression (Fig. 3b, c). Next, we pretreated KYSE-170 cells with cell-permeable octyl-D- or L-2-HG followed by addition of erastin. Both enantiomers of 2-HG promoted erastin-induced ferroptosis in KYSE-170 cells (Fig. 3d, e). Although D-2-HG showed higher potency in promoting ferroptosis than L-2-HG when used at the same concentration, we found that L-2-HG accumulation within cells was only about 10% of D-2-HG after 24 h treatment (Fig. 3f). In accordance, cell-permeable octyl-D-2-HG promoted HT-1080(*IDH1*^+/−^) cells’ sensitivity to erastin (Fig. 3g, h). Consistent with their role in promoting ferroptosis, D-2-HG caused a stronger lipid ROS accumulation (Fig. 3i). Based on these data, we conclude that D-2-HG serves as the effector of mutant *IDH1* within cells to enhance cells’ sensitivity to ferroptosis.

### Mutant IDH1 exacerbates erastin-induced glutathione depletion

**Fig. 4:**
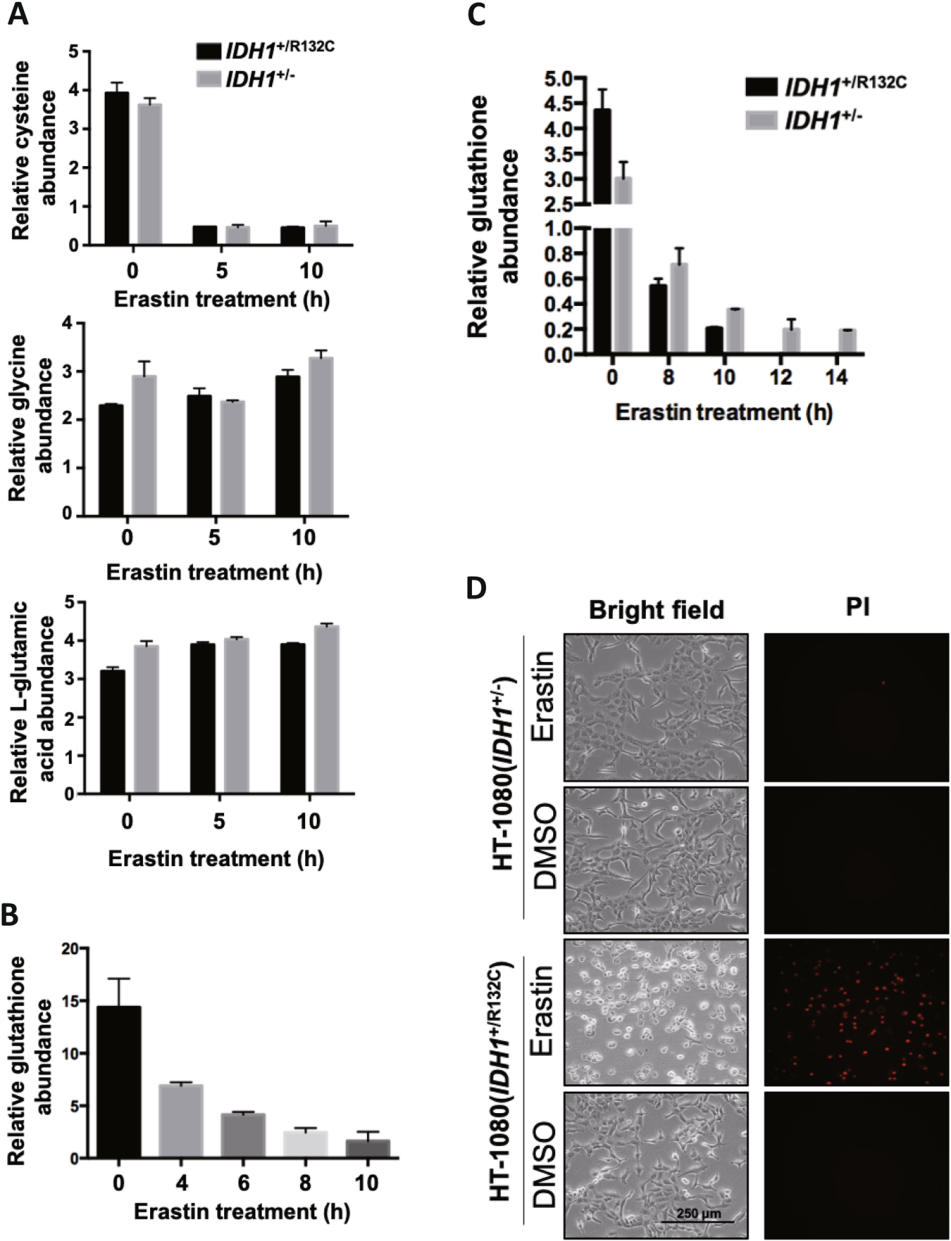
Mutant IDH1 exacerbates erastin-induced glutathione depletion. **a** Measurement of cellular levels of glycine, cysteine and L-glutamic acid. HT-1080(*IDH1*^+/R132C^) or HT-1080(*IDH1*^+/−^) cells were treated with erastin for indicated time and cellular glycine, cysteine and L-glutamic acid levels were determined by GC-MS. **b** Erastin treatment reduces cellular glutathione level over time. HT-1080(*IDH1*^+/R132C^) cells were treated with erastin for indicated time and cellular glutathione levels were measured using Total Glutathione Assay Kit. **c** Mutant IDH1 exacerbates erastin-induced glutathione depletion. HT-1080(*IDH1*^+/R132C^) or HT-1080(*IDH1*^+/−^) cells were treated with erastin (10 μM) for indicated time and cellular glutathione levels were measured using Total Glutathione Assay Kit. **d** HT-1080(*IDH1*^+/R132C^) or HT-1080(*IDH1*^+/−^) cells were treated with erastin for 12 h and cells were microscopically photographed

Erastin-induced ferroptosis through inhibiting cystine uptake and thus decreasing cellular concentration of cysteine which is the substrate for glutathione synthesis^30^. Moreover, reduced glutathione (GSH) is indispensable for the enzymatic activity of GPX4^31^. To test whether IDH1^R132C^ mutant influences erastin-induced exhaustion of glutathione, we determined the cellular levels of cysteine, glycine, and L-glutamic acid using GC-MS. After treatment with erastin for 5 h, cellular cysteine level decreased to about ten percent of that at steady state, and then it maintained at this level up to 10 h after erastin treatment (Fig. 4a). Glycine and glutamic acid, the other two amino acids used for glutathione synthesis, remained stable in response to erastin treatment. The reduction of cysteine showed no difference between HT-1080(*IDH1*^+/R132C^) and HT-1080(*IDH1*^+/−^) cells after erastin treatment, indicating the cysteine reduction is unlikely to be the direct cause for the different sensitivity to erastin between these two cell lines.

Next, we measured the endogenous glutathione level. During the early phase (0–4 h) after erastin addition, cellular glutathione level dropped by about 50%. Then the endogenous glutathione level continued to drop during late phase (6–10 h) after erastin addition (Fig. 4b). We found the basal level of glutathione in HT-1080(*IDH1*^*+/*−^) cells was lower than that in HT-1080(*IDH1*^+/R312C^) cells. Erastin treatment caused a decline of glutathione level in both HT-1080(*IDH1*^+/R132C^) and HT-1080(*IDH1*^+/−^) cells, but notably the rate of decline was much lower in HT-1080(*IDH1*^+/−^) cells (Fig. 4c). Glutathione level was not detectable after 12 h in HT-1080(*IDH1*^+/R132C^) cells as most cells died then (Fig. 4d). Erastin-induced ferroptosis is thought as a process resulted from gradually decreasing glutathione which leads to the accumulation of lipid ROS. Our results suggest the expression of mutant IDH1 may accelerate the glutathione depletion and lipid ROS accumulation, and thereby promote ferroptosis.

### IDH1 mutation reduces GPX4 protein level

**Fig. 5:**
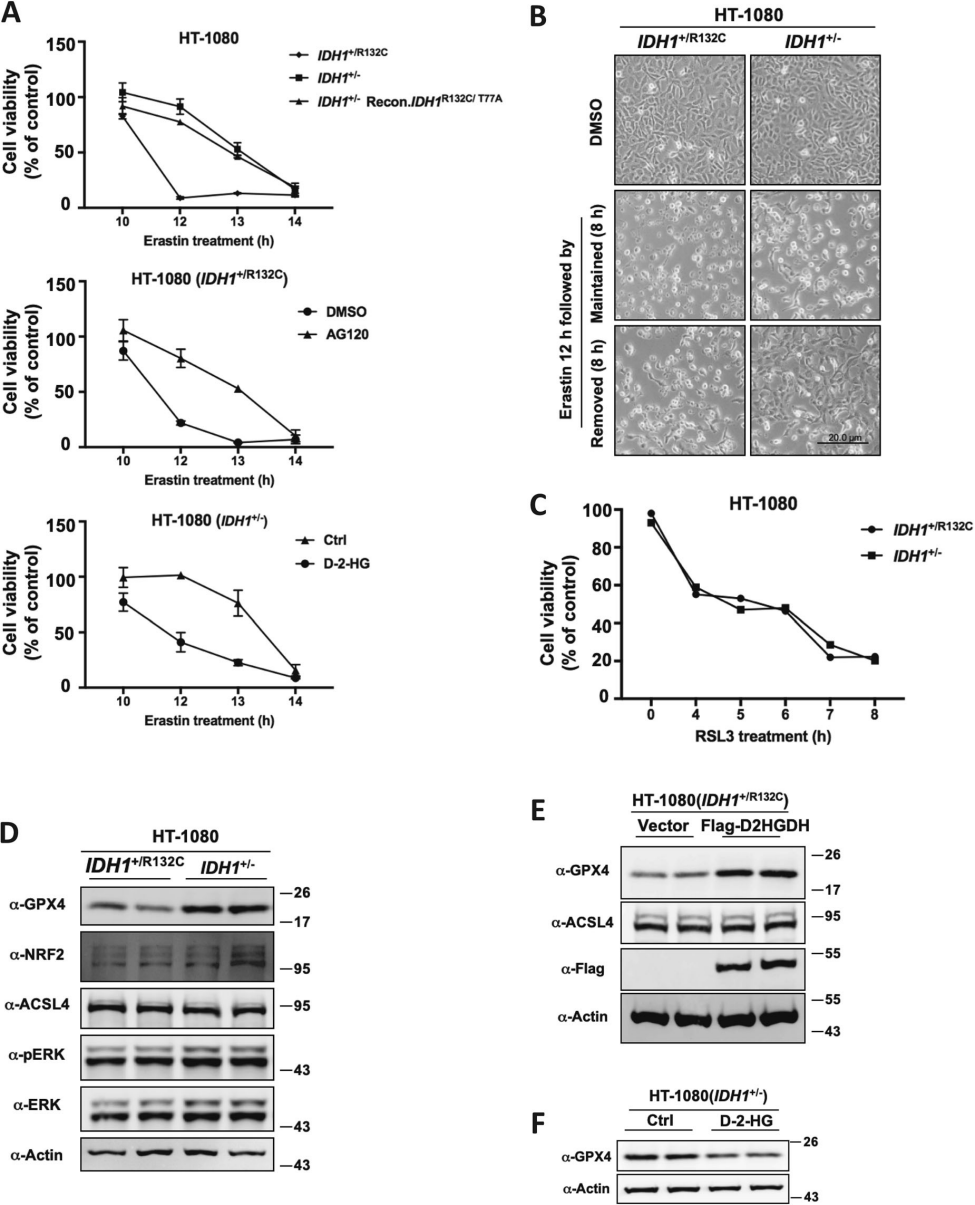
IDH1 mutation reduces GPX4 protein level. **a** IDH1^R132C^ mutation and 2-HG treatment accelerate the onset of erastin-induced ferroptosis. HT-1080 cells with indicated genotypes or treatment were treated with erastin (10 μM) for indicated time, and cell viability were assayed by trypan blue staining. **b** Ferroptosis is a reversible process before reaching a point of no-return. HT-1080(*IDH1*^+/R132C^) or HT-1080(*IDH1*^+/−^) cells were treated with erastin for 12 h and then erastin was either removed from or kept in the culture medium for additional 8 h, followed by microscopic photograph. **c** Mutant IDH1 does not enhance ferroptosis in cells treated with GPX4 inhibitor RSL3. HT-1080(*IDH1*^+/R132C^) or HT-1080(*IDH1*^+/−^) cells were treated with RSL3 (2 μM) for indicated time, and cell viability were assayed by trypan blue staining. **d** Knocking out of IDH1R132C allele up-regulates the protein level of GPX4. The protein levels of Nrf2, Acsl4, Erk, p-Erk, GPX4, and Actin in HT-1080(*IDH1*^+/R132C^) or HT-1080(*IDH1*^+/−^) cells were detected by western blot. **e** D2HGDH overexpression upregulates the protein level of GPX4. The protein level of Acsl4, Flag-D2HGDH, GPX4 and Actin in HT-1080(*IDH1*^+/R132C^) cells expressing vector or Flag-D2HGDH were detected by western blot. **f** D-2-HG treatment down-regulates the protein level of GPX4. The protein level of GPX4 and Actin in HT-1080(*IDH1*^+/−^) cells with or without D-2-HG treatment were detected by western blot

The above data establish a role of IDH1^R132C^ mutant and its product D-2-HG in enhancing ferroptosis. However, time-course experiments demonstrated that suppression of IDH1^R132C^ by deletion of mutant allele or inhibitor treatment slowed but did not abolish ferroptosis (Fig. 5a). Interestingly, we also observed that if erastin was removed after 12 h treatment, HT-1080(*IDH1*^+/R132C^) cells continue to undergo ferroptosis while HT-1080(*IDH1*^+/−^) cells survived (Fig. 5b). These data indicate that ferroptosis is a reversible process until a critical point is reached.

In searching for the mechanism underlying the effect that mutant IDH1 enhance ferroptosis, we found, surprisingly, that unlike the response to erastin, HT-1080(*IDH1*^+/R132C^) and HT-1080(*IDH1*^+/−^) cells showed no difference to the treatment of RSL3, a direct inhibitor of GPX4 that rapidly induces ferroptosis (Fig. 5c). This result suggests that mutant IDH1 may enhance ferroptosis through targeting GPX4. GPX4 is the only known enzyme in mammalian cells that can eliminate lipid ROS. We found that the protein level of GPX4 is up-regulated when *IDH1*^R132C^ was deleted (Fig. 5d). We confirmed the effect of mutant IDH1 on GPX4 protein level by the observation that overexpression of D2HGDH in HT-1080(*IDH1*^+/R132C^), which cleared out D-2-HG produced by mutant IDH1, upregulated GPX4 (Fig. 5e). Conversely, treatment of HT-1080(*IDH1*^+/−^) cells with cell-permeable D-2-HG downregulated the protein level of GPX4 (Fig. 5f). These observations reveal a possible molecular mechanism that IDH1^R132C^ mutation sensitizes cells to erastin-induced ferroptosis through regulating the protein level of GPX4.

## Discussion

Since ferroptosis was defined in 2012^3^, much effort has been devoted to elucidate the mechanisms underlying this new form of regulated cell death. Several major aspects involved in ferroptosis regulation have been identified, including iron metabolism, lipid oxidation, and redox homeostasis^32^. However, a key unanswered question is why different cell lines exhibit markedly different sensitivities to the same ferroptosis signals. This suggests that ferroptosis is highly cell-context dependent and is likely affected by different genetic backgrounds. In this study, we discover that the *IDH1* mutation borne by HT-1080 cells, a model cell line commonly used for ferroptosis studies, contributes to the high sensitivity of HT-1080 cells to ferroptosis-inducing agent. Deletion of the mutant *IDH1* allele rendered cells less sensitive to erastin-induced ferroptosis, and conversely, re-expression of mutant, but not WT IDH1 re-sensitized HT-1080(*IDH1*^+/−^) cells to ferroptosis. We further demonstrated that D-2-HG, the product of mutant IDH1, mediates the effect of mutant IDH1 in sensitizing HT-1080 cells to ferroptosis. In accordance, inhibitors of mutant IDH1 or overexpression of D2HGDH, reduced cells’ sensitivity to erastin-induced ferroptosis. These findings uncover a previously unrecognized activity of IDH mutation in promoting ferroptosis. It was recently reported that that inhibition of mitochondrial TCA cycle or electron transfer chain (ETC) reduces lipid peroxide accumulation and ferroptosis^33^. Our study adds another important evidence supporting the notion that ferroptosis may be enhanced by altered metabolism linked to pathological mutation.

Mutant IDH1/2 gains a neomorphic activity of converting α-KG to 2-HG. Two other metabolites, fumarate, and succinate, are found to accumulate in cells with mutations in succinate dehydrogenase (SDH) or fumarate hydratase (FH). One common mechanism underlying these three metabolites is that these oncometabolites, because of their structural similarity with α-KG, promote cell transformation through acting as an antagonist of α-KG and competitively inhibiting α-KG-dependent dioxygenases, such as histone and DNA demethylases. Interestingly, a recent study reported that *FH* mutation renders cells more resistant to cysteine-starvation induced ferroptosis^34^. The authors suggest that loss of FH activity accumulates fumarate and disrupts TCA cycle, leading to cell resistance to ferroptosis. In contrast, our study found IDH1 mutation, which accumulates oncometabolite D-2-HG, increases cells’ sensitivity to erastin-induced ferroptosis. Hence, D-2-HG affects ferroptosis differently from fumarate, suggesting the possibility that these two metabolites may affect ferroptosis via a mechanism independent of inhibiting an α-KG-dependent dioxygenase(s). For example, IDH mutation has been reported to cause respiration inhibition through inducing hypersuccinylation in the mitochondria^34^. Since normal TCA cycle is essential for ferroptosis^33^, we speculate that 2-HG sensitizes cell to ferroptosis through a mechanism linked to mitochondrial function. Indeed, we found IDH1 mutation leads to more rapid accumulation of lipid ROS, which is believed to be the key inducer of ferroptosis. Impaired glutathione homeostasis may contribute to this phenomenon, evidenced by a faster exhaustion of glutathione in HT-1080(*IDH1*^+/R132C^) cells upon erastin treatment (Fig. 4c).

Notably, IDH1 mutation or 2-HG treatment leads to downregulation of GPX4 protein level and elimination of mutant IDH1 or overexpression of D2HGDH upregulated GPX4 protein level. Interestingly, we further observed that IDH1 mutation does not affect RSL3 induced ferroptosis (Fig. 5c), supporting the notion that IDH1 mutation sensitize cells to ferroptosis through GPX4. RSL3 inhibits GPX4 in a concentration-dependent manner. It is notable that previous studies showed cells with GPX4 overexpression by several folds were less sensitive to RSL3^30^. In our experiment, however, high 2-HG level decreased GPX4 protein level by about fifty percent (Fig. 5d). In this case, the inhibitory effect of RSL3 on GPX4 would be exacerbated. We used RSL3 at a concentration of 2 μM, which is generally used for inhibiting GPX4 and rapidly inducing ferroptosis in HT-1080 cells, to demonstrate that full suppression of GPX4 made cells insensitive to 2-HG, indicating that IDH1 mutation or 2-HG accumulation may affect ferroptosis through regulating GPX4. GPX4 is thus far the only known enzyme in mammalian cells to eliminate lipid ROS and thus its expression level and activity determines cell fate upon ferroptotic signals. It is conceivable that IDH1 mutation and D-2-HG regulate the expression of GPX4.

In addition to monotherapy targeting mutant IDH enzymes through pharmacological inhibition, multiple combination therapies are being explored to target the vulnerability of IDH-mutant tumors. These include increased sensitivity of IDH-mutant tumors to alkylating agents^35,36^, to inhibitors of nicotinamide phosphoribosyltransferase (NAMPT)^37^, and inhibitor of poly ADP ribose polymerase (PARP)^38^. Our findings suggest that sensitizing cells to ferroptosis may be worth to explore for the treatment of IDH-mutant tumors.
